# Notification that new names of prokaryotes, new combinations and new taxonomic opinions have appeared in volume 75, part 7 of the IJSEM

**DOI:** 10.1099/ijsem.0.006893

**Published:** 2025-10-31

**Authors:** Aharon Oren, Markus Göker

**Affiliations:** 1 The Institute of Life Sciences, The Hebrew University of Jerusalem, The Edmond J. Safra Campus, 9190401 Jerusalem, Israel; 2 Leibniz Institute DSMZ – German Collection of Microorganisms and Cell Cultures, Inhoffenstrasse 7B, 38124 Braunschweig, Germany

This listing of names of prokaryotes published in a previous issue of the *International Journal of Systematic and Evolutionary Microbiology (IJSEM)* is provided as a service to microbiology to assist in the recognition of new names and new combinations. This procedure was proposed by the Judicial Commission [1]. The names given herein are listed according to the Rules of priority (i.e. time and date of publication of the articles on the journal’s platform and the order of valid publication of names in the original articles) [2].

**Table:** 

**Order** of article publication	Name/authors	Proposed as	Article no.	Page no.
1	*Nocardiopsis protaetiae* Liu *et al*. 2025	sp. nov.	006832	6
2	*Oceaniferula spumae* Huang *et al*. 2025	sp. nov.	006828	8
3	*Legionella sheltonii* Sauerborn Klobucar *et al*. 2025	sp. nov.	006813	11
4	*Streptococcus handemini* Pu *et al*. 2025	sp. nov.	006814	9
4	*Streptococcus jiangjianxini* Pu *et al*. 2025	sp. nov.	006814	9
4	*Streptococcus tangpeifui* Pu *et al*. 2025	sp. nov.	006814	10
4	*Streptococcus huangxiaojuni* Pu *et al*. 2025	sp. nov.	006814	10
5	*Aestuariibius violaceus* Lee *et al*. 2025	sp. nov.	006834	7
6	*Chlamydia vaughanii* Marquis *et al*. 2025	sp. nov.	006753	10
7	*Fodinibius alkaliphilus* He *et al*. 2025	sp. nov.	006840	7
7	*Fodinibius salipaludis* He *et al*. 2025*	sp. nov.	006840	9
8	*Brachymonas wangyanguii* Gao *et al*. 2025	sp. nov.	006837	7
9	*Methanosuratincola* Wu *et al*. 2025	gen. nov.	006839	5
9	*Methanosuratincola petrocarbonis* Wu *et al*. 2025	sp. nov.	006839	5
9	*Methanosuratincolaceae* Wu *et al*. 2025	fam. nov.	006839	6
9	*Methanosuratincolales* Wu *et al*. 2025	ord. nov.	006839	6
9	*Methanosuratincolia* Wu *et al*. 2025	class. nov.	006839	6
10	*Corynebacterium rhinophilum* Popowitch *et al*. 2025	sp. nov.	006835	8
11	*Thermohahella* Ganbat *et al*. 2025	gen. nov.	006843	8
11	*Thermohahella caldifontis* Ganbat *et al*. 2025	sp. nov.	006843	8
12	*Pseudomonas alabamensis* Fullem *et al*. 2025	sp. nov.	006848	8
13	*Agromyces chitinivorans* Xu *et al*. 2025	sp. nov.	006831	7
13	*Agromyces litoreus* Xu *et al*. 2025	sp. nov.	006831	7
13	*Agromyces zhanjiangensis* Xu *et al*. 2025	sp. nov.	006831	8
14	*Fibrella aquatica* Baek *et al*. 2025	sp. nov.	006842	8
14	*Fibrella musci* Baek *et al*. 2025	sp. nov.	006842	10
14	*Fibrella arboris* Baek *et al*. 2025	sp. nov.	006842	10
15	*Herbiconiux liangxiaofengii* Pu *et al*. 2025	sp. nov.	006845	9
15	*Herbiconiux wuyangfengii* Pu *et al*. 2025	sp. nov.	006845	9
15	*Herbiconiux liukaitaii* Pu *et al*. 2025	sp. nov.	006845	9
16	*Pseudidiomarina salilacus* Yu *et al*. 2025	sp. nov.	006846	7
17	*Nioella halotolerans* Han *et al*. 2025	sp. nov.	006858	7
18	*Pseudogemmobacter sonorensis* Lange-Enyedi *et al*. 2025	sp. nov.	006859	9
19	*Streptosporangium soli* Yin *et al*. 2025	sp. nov.	006853	7
20	*Alitibacter* Bisgaard and Christensen 2025	gen. nov.	006867	4
20	*Alitibacter langaaensis* (Mutters *et al*. 1985) Bisgaard and Christensen 2025	comb. nov. [basonym: *Pasteurella langaaensis* corrig. Mutters *et al*. 1985]	006867	4
21	*Macrococcus equi* Belhout *et al*. 2025	sp. nov.	006861	7
21	*Macrococcus animalis* Belhout *et al*. 2025	sp. nov.	006861	9
22	*Streptomyces antarcticus* Sahin *et al*. 2025	sp. nov.	006856	13

*Effectively published as *Aliifodinibius salipaludis* Zhao *et al*. 2020.

The following taxonomic opinions (i.e. the emendation of circumscriptions or the creation of synonyms) do not affect the status of a name under the International Code of Nomenclature of Prokaryotes. These taxonomic opinions can neither be considered as approved nor as disapproved by the International Committee on Systematics of Prokaryotes.

**Table:** 

Name/authors:	Article no.	Page no.
*Aestuariibius* Park *et al*. 2018 emend. Lee *et al*. 2025	006834	7
*Blautia coccoides* (Kaneuchi *et al*. 1976) Liu *et al*. 2008 pro synon. *Blautia producta* (Prévot 1941) Liu *et al*. 2008	006823	6
*Geomonas nitrogeniifigens* Liu *et al*. 2022 pro synon. *Geomonas diazotrophica* Xu *et al*. 2022	006841	8
*Geomonas fuzhouensis* Yang *et al*. 2023 pro synon. *Geomonas subterranea* Liu *et al*. 2022	006841	8
